# Sudachi peel extract powder including the polymethoxylated flavone sudachitin improves visceral fat content in individuals at risk for developing diabetes

**DOI:** 10.1002/fsn3.2339

**Published:** 2021-06-15

**Authors:** Yasuhiro Shikishima, Rie Tsutsumi, Ayuka Kawakami, Hiroyuki Miura, Yoshitaka Nii, Hiroshi Sakaue

**Affiliations:** ^1^ Ikeda Yakusou Co. Ltd Tokushima Japan; ^2^ Department of Nutrition and Metabolism Institute of Biomedical Sciences Tokushima University Tokushima Japan; ^3^ Food and Biotechnology Division Tokushima Industrial Technology Center Tokushima Japan

**Keywords:** *Citrus sudachi*, glucose metabolism, lipid profile, randomized clinical trial, visceral fat

## Abstract

In vitro and animal studies have indicated that extracts from the peel of the Japanese *Citrus* *sudachi*, including sudachitin, ameliorate hyperlipidemia and reduce obesity. Sudachitin, a polymethoxylated flavone, has been reported as having favorable effects on lipid and glucose metabolism but results from clinical trials have been inconsistent. The aim of this study was to determine the effect of consuming capsules of sudachi peel extract powder on visceral fat in Japanese men and women in a randomized controlled trial. This was a 12‐week randomized, double‐blind, placebo‐controlled trial involving 41 participants aged 30–65 years with BMI 23–30 kg/m^2^, randomly allocated to receive either sudachi peel extract powder (sudachitin 4.9 mg/day, *n* = 21) or placebo (*n* = 20) of identical appearance. The primary outcome measure was visceral fat mass, assessed during intervention. Thirty‐eight of the 41 subjects completed the protocol. Compared with placebo, sudachi peel extract powder significantly reduced the ratio of visceral fat to subcutaneous fat, and moderately reduced waist circumference, a metabolic syndrome marker. Glycemic control and lipid profile were not changed significantly in these subjects. Consumption of capsules of sudachi peel extract powder favorably improves the ratio of visceral fat to subcutaneous fat in individuals at risk for developing diabetes, especially in individuals with large visceral fat area, while not adversely affecting glycemic control.

## Graphical abstract

Extracts of Citrus sudachi (sudachi) peel, containing the polymethoxylated flavone sudachitin, have shown promise in vitro and in animal models. Compared with placebo, sudachi peel extract significantly reduced the ratio of visceral fat to subcutaneous fat (−0.0265%, *p* = .0485), and moderately reduced visceral fat and waist circumference.

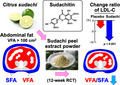

**Strengths and limitations of this study**

- A randomized, double‐blind clinical trial was performed to investigate the benefits of sudachi peel extract powder.
- Intake of sudachi peel extract powder improved the ratio of visceral fat to subcutaneous fat.
- As limitation, we only found favorable changes in the ratio of visceral fat to subcutaneous fat, but not in other markers.

## 1 INTRODUCTION

The rising incidence of diabetes worldwide during the past four decades has become a significant global public health challenge. The global prevalence of diabetes was estimated at 8% in 2013, and increases have been particular prominent in Asian countries such as China and India. (Wang et al., 2017) There is a large burden of diabetes in Japan, where 12% of adults have diabetes of which 76.8% receive medical care. (Urakami et al., 2019) Diabetes management is very cost‐effective when initiated at an early stage. (Urakami et al., 2019) Dietary and lifestyle modifications are recommended as the primary strategies for type 2 diabetes prevention. (Li et al., 2008; Perreault et al., 2012) The inclusion of dietary phytochemicals is considered a feasible and practical strategy.

Flavonoids are responsible for the green to yellow pigments found in plants (fruits, vegetables, flowers, and grains), and a subgroup of these is found in the fruit of *Citrus* *sudachi* (sudachi). Results from our previous in vitro studies indicated that extract of sudachi peel may decrease hyperglycemia and improve lipid profile through activation of adenosine monophosphate‐activated protein kinase (Xu et al., 2018) or by regulating transcriptional factor forkhead box protein O1, both of which are involved in the molecular mechanisms underlying critical signaling pathways in glycolipid metabolism.

Several small‐scale randomized controlled trials (RCTs) have tested the effects of sudachi extract or sudachitin‐rich extracts on glucose homeostasis and insulin sensitivity, but findings were inconsistent. One study showed attenuation of hyperglycemia and/or hyperlipidemia, (Akaike et al., 2014) while another showed ineffective results. A previous pilot study indicated that sudachi peel has the potential to safely improve abdominal obesity and to lower serum levels of triglycerides (TG) in obese individuals with hypertriglyceridemia. These data indicate that purified sudachi peel could improve insulin resistance, dyslipidemia, and antioxidant capacity. However, whether these effects could be extended to individuals at risk for developing diabetes is unclear. Since we had previously performed safety and toxicological evaluation of a novel concentrated sudachi extract in the form of a powder, we proposed this RCT to examine its effects on metabolic profiles in people with individuals at risk for developing diabetes to determine whether this intervention would be efficacious for the primary prevention of diabetes.

## 2 RESEARCH DESIGN AND METHODS

### 2.1 Patient and public involvement

Our research question was that if citrus sudachi extract improves metabolic profile and body fat composition especially visceral fat. The primary outcomes were epidermal and subcutaneous fat assessed by computed tomography (CT) scan and waist circumference. The secondary outcome was a lipid profile including total cholesterol (TC), triglycerides (TG), high‐density lipoprotein (HDL), and low‐density lipoprotein (LDL) cholesterol. Participants were Japanese men and women aged 30–65 years with mild overweight, BMI 23–30 kg/m^2^. Participants were excluded if they had a medical history of diabetes, untreated thyroid disease, polycystic ovarian syndrome, serious liver or kidney dysfunction, were currently suffering from acute or chronic infectious diseases or traumatic injury or surgery, or were currently taking or in the preceding 6 months had taken hypoglycemic or weight reduction agents, were using glucocorticoids, were lactating or pregnant women, or had a known allergy to citrus or flavonoids. No patients were involved in this study. Participants were recruited from local communities in Kagawa Prefecture, Japan, through advertising flyers, medical record review, or clinicians’ recommendations at outpatient clinics. Potential participants were interviewed by trained research staff over the telephone or in person with a structured screening questionnaire. The results were not disseminated to study participants. The burden of the intervention was assessed by participants themselves. Potential participants were interviewed by trained research staff over the telephone or in person with a structured screening questionnaire. Participants with a recent medical record of hyperglycemia were invited to attend a clinical visit to undergo anthropometric assessment for confirmation of eligibility. The enrollment and intervention were conducted at a local hospital from October 2018 to April 2019. The Ethics Committee of Linking Setouchi Innate immune network approved the study protocol (20180919). All participants signed written informed consent prior to enrollment. The trial was registered at the University hospital Medical Information Network (Japan).

### 2.2 Study design, intervention, randomization, and blinding

This was a 12‐week CONSORT (Consolidated Standards of Reporting Trials)‐compliant, randomized, double‐blind, placebo‐controlled trial. A total of 88 participants were recruited, and 41 eligible participants with overweight were enrolled and randomly assigned to two intervention arms to receive either purified sudachi extract (1,050 mg/day including 4.9 mg sudachitin) or placebo. The dosage was determined based on a previous clinical trial. (Akaike et al., 2014) Our previous pilot study indicated that dairy intake of 5 mg of sudachi peel has the potential to safely improve abdominal obesity.

Sequence generation was performed by a third party with a computer program “Mujinwari” (Iruka system Co. Ltd.). Research staff were not involved in the randomization or the labeling work. The serial numbers and the corresponding supplements were assigned to the eligible subjects in the order of final enrollment into the trial. Participants, investigators, and laboratory technicians were blinded to the treatment assignments until the conclusion of the trial. The stratification analysis was performed by the stratified substitution block method using three factors, sex, triglyceride, and BMI, as stratification factors.

### 2.3 Supplement preparation

The sudachi peel extract powder and placebo capsules were produced by the Ikeda Yakusou Co. Ltd. The sudachi peel extract powder contained high concentrations of sudachitin (1.4%, 4.9 mg/day), which comprises several different natural flavonoids purified from sudachi peel (see File S1 for ingredients). Dairy intake in the sudachi peel group was equivalent to 1/3 of a whole Citrus sudachi. The sudachi capsules contained sudachi peel extract powder and γ‐cyclodextrin and were included to maintain the stability of flavonoids; the placebo capsules contained only γ‐cyclodextrin. Participants were instructed to consume 3 capsules daily, preferably 30 min after a meal. They were asked to maintain their usual dietary intakes and physical activities. They were required not to use any supplements containing flavonoids and to avoid consumption of sudachitin‐rich foods (sudachi peel, etc.) during the intervention period. Supplements were delivered to subjects every 2 weeks after randomization.

### 2.4 Data collection

Information about the individual participants was collected by trained research staff via face‐to‐face interview based on a structured questionnaire covering medical history, uses of medications, dietary habits, and physical activities. Overnight fasting (8–10 hr) venous blood samples were collected between 8:00 and 9:00 a.m., and other assessments were performed at both baseline and at the end of treatment. Baseline and follow‐up specimens from the same individuals were measured in one batch to minimize inter‐assay variability.

### 2.5 Primary and secondary outcome measures

The primary outcome was umbilicus visceral fat area assessed by computed tomography (CT) scan. The secondary outcomes are umbilicus subcutaneous fat area, physical examination data (height, weight, body fat percentage, BMI, and waist circumference), and lipid profile including total cholesterol (TC), TG, high‐density lipoprotein (HDL), and low‐density lipoprotein (LDL) cholesterol. The fasting glucose, HbA1c, fasting insulin, and uric acids were also assessed. The homeostasis model assessment of insulin resistance (HOMA‐IR) and β‐cell function (HOMA‐β) was calculated based on fasting glucose (FG) and fasting insulin (FIns).

HOMA‐IR = FIns (mU/mL) × FG (mmol/L)/22.5,
HOMA‐β = FIns × 20/(FG −3.5).

Biomarkers of liver and kidney function were measured for safety assessment, including serum alanine transaminase (ALT), aspartate aminotransferase (AST), and creatinine. All of the biomarkers were measured at both baseline and at the end of the 12‐week treatment.

### 2.6 Dietary intake, physical activity, and anthropometric assessments

Assessments of dietary intake were based on 3‐day food records that were completed by subjects and checked by investigators at baseline and at the end of the trial. Dietary intakes of nutrients were calculated according to the Japanese Food Composition Table. Participants were queried about the frequency and intensity of habitual physical activities before and after the intervention. Bodyweight, height, waist and hip circumference, and blood pressure were measured according to standard protocols.

### 2.7 Compliance assessment

Every 2 weeks, participants were requested to attend a general visit when they were asked to return any unused capsules and were given new capsules for the next interval. At each regular visit, patients were specifically asked about adverse events. An adverse event was defined as any discomfort during the intervention. Compliance was assessed by counting the unused capsules at each visit. Good compliance was defined as consuming more than 80% of the provided capsules and completing all assessments and sample collections.

### 2.8 Sample size planning

Sample size estimation was based on a previous clinical pilot study using sudachi capsules as a treatment [Y. Shikishima, unpublished data], in which the changes in standard deviation (*SD*) for waist circumference and TG were 0.2% (*SD* 0.4%). A sample size of 15 per group would provide 80% power to detect a significant change in waist circumference or TG given a change of 5%, using a conventional assumption of a two‐tailed α level of 0.05. Allowing for a 20% drop‐out rate, we planned to recruit 20 subjects per group.

### 2.9 Statistical analysis

Statistical analysis was performed using JMP (JMP SAS Inc.). Baseline characteristics were compared between the two intervention groups to determine their baseline comparability. Data were analyzed according to an intention‐to‐treat (ITT) principle, which included all 38 randomized subjects. Secondary analyses comprised a per‐protocol (PP) analysis which included subjects exhibiting good compliance and excluded subjects who withdrew or who exhibited poor compliance.

The “last observation carried forward” method was used for processing the missing values in the ITT analysis, while the PP analysis did not use any imputation method. The net changes at 12 weeks in outcome variables between the two study groups were compared by both *t* test and analysis of covariance (ANOVA) with adjustment of baseline parameters (baseline value, age, sex, and medications for lowering blood pressure and lipids).

Stratification analyses were conducted to test whether the effect of sudachi peel differed across various subgroups including subjects with triglycerides (TG) > 140 mg/dl, HbA1c > 6.0%, VFA >100 cm^2^, or waist circumference >100 cm. Before the subgroup analyses, we tested the effect modification by adding an interaction term of treatment and subgroup variables to the univariate models.

## 3 RESULTS

### 3.1 Subject characteristics at baseline and follow‐up

**FIGURE 1:**
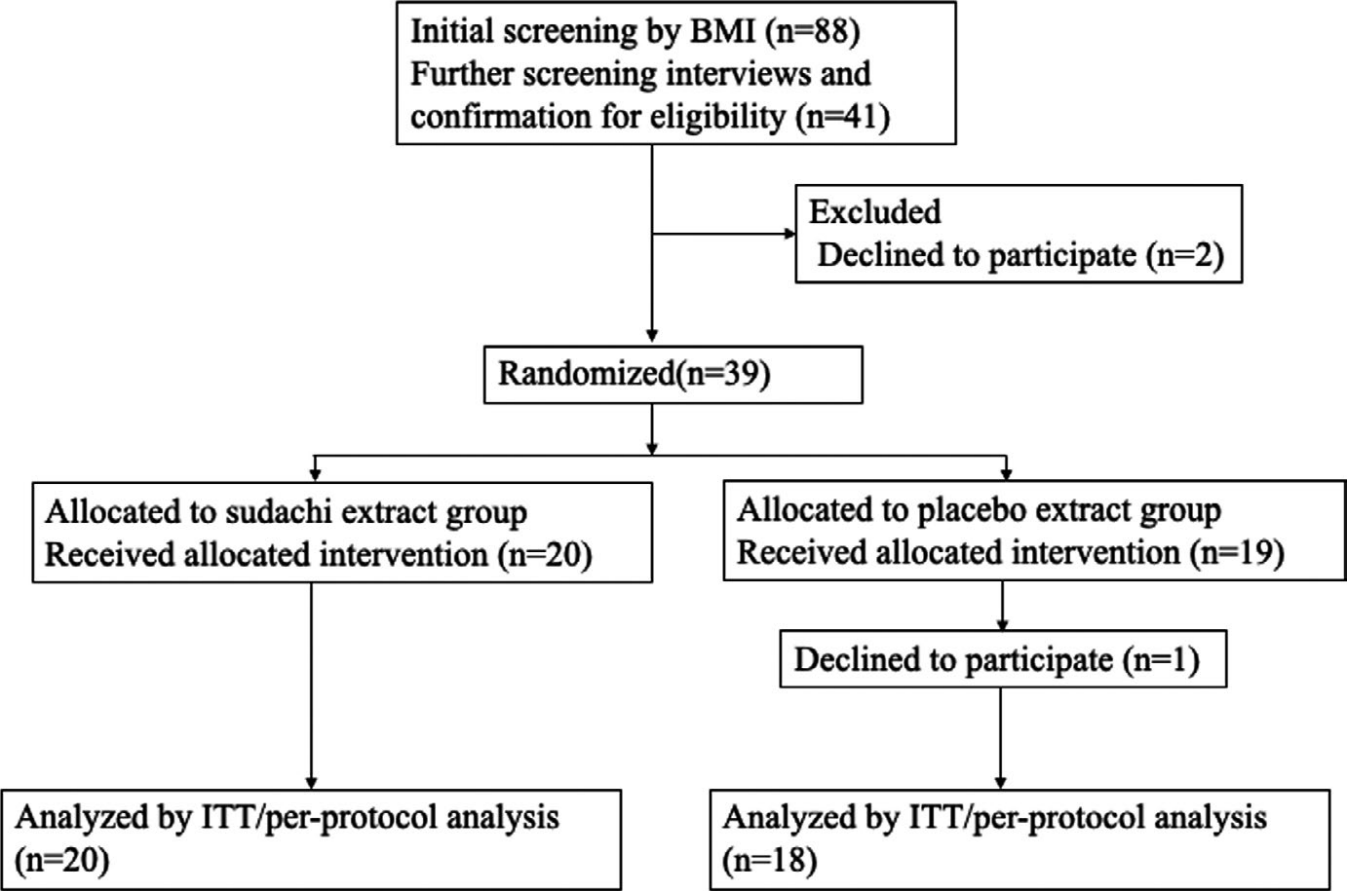
The CONSORT flowchart of the study

A total of 38 participants were randomly allocated into two study arms. Among the 41 participants, 2 (4.9%) withdrew from the study before the intervention and one (2.4%) during follow‐up. No significant difference was observed in the proportion of valid completers among the two groups. As shown in Figure 1, a total of 38 subjects attended the final visit and received the assessment. Baseline measurements were performed before the randomization. Participants in the two groups were comparable in terms of age, sex, education, medical history, bodyweight, BMI, waist circumference, and systolic and diastolic blood pressure (BP). Smoking, habitual alcohol drinking, sports activity, and dietary intake of total energy and nutrients did not differ between these groups (data not shown). No significant difference was observed between the two groups in anthropometric markers and dietary intake of nutrients and flavonoids in either the baseline or the follow‐up data (data not shown).

### 3.2 Effects of sudachi extract on glycemic markers and lipid profile

The baseline parameters for epidermal and subcutaneous fat were comparable between the two study groups (Table 1). As shown in Table 2, HbA1c was somewhat improved in the sudachi extract group (*p* = .066). There were no significant changes in glycemic control or lipid profile. Safety markers, such as inflammation including liver and renal function including ALT, AST, and creatinine, did not differ between the two groups.

**TABLE 1:** Characteristic of subjects at baseline

	Sudachi extract	Placebo	*p*
	(*n* = 20)	(*n* = 18)	
Demographics			
Age (year)	46.5 ± 9.1	46.8 ± 8.5	.920
Gender (male/female)	10/10	9/9	
Anthropometrics			
Weight (kg)	69.0 ± 9.5	68.7 ± 9.7	.953
BMI (kg/m^2^)	25.0 ± 2.1	25.5 ± 2.2	.465
Body fat (%)	29.5 ± 6.2	30.7 ± 6.9	.539
Waist circumference (cm)	89.7 ± 6.2	91.8 ± 5.1	.354
Blood pressures (BP, mm Hg)			
Systolic BP	134.1 ± 19.4	134.0 ± 15.8	.884
Diastolic BP	80.2 ± 10.8	82.4 ± 9.9	.558
Hypertension (>140/90 mm Hg)	3	4	*n*.s
Heart rate	71.2 ± 10.3	71.1 ± 11.2	.895
CT: fat area (cm^2^)			
TFA (cm^2^)	243.7 ± 63.9	241.2 ± 72.8	.107
SFA (cm^2^)	161.6 ± 49.5	186.7 ± 51.3	.627
VFA (cm^2^)	82.1 ± 32.3	94.6 ± 29.7	.272
VFA/SFA	0.56 ± 0.04	0.55 ± 0.05	.843

**TABLE 2:** Change of glycemic and lipid profile

	Sudachi			Placebo			*t*‐test
	Baseline	12 weeks	Change	Baseline	12 weeks	Change	
Markers on glycemic control							
HbA1c (%)	5.56 ± 0.27	5.51 ± 0.28	−0.05	5.69 ± 0.51	5.65 ± 0.47	0.039	.0660
Fasting glucose (mg/dl)	91.80 ± 9.19	91.95 ± 10.45	0.15	91.44 ± 9.19	92.11 ± 6.93	0.67	.3048
Fasting insulin (uU/ml)	6.01 ± 2.14	5.89 ± 3.11	−0.21	7.21 ± 3.13	7.21 ± 3.13	0.017	.6178
HOMA IR	1.40 ± 0.12	1.38 ± 0.17	−0.15	1.64 ± 0.09	1.64 ± 0.09	0.002	.8966
HOMA β	79.90 ± 7.65	77.04 ± 6.54	−2.87	98.76 ± 5.87	98.76 ± 5.87	−8.73	.5340
Lipid profile							
Triglyceride (mg/dl)	104.02 ± 44.21	113.6 ± 66.36	9.62	131.77 ± 31.24	172.88 ± 45.32	41.1	.5976
Total cholesterol (mg/dl)	220.45 ± 32.57	215.55 ± 38.73	−4.00	211.47 ± 35.87	207.04 ± 35.83	−4.01	.8116
HDL cholesterol (mg/dl)	60.15 ± 14.59	60.65 ± 15.67	0.22	59.67 ± 13.77	59.88 ± 14.85	0.22	.8108
LDL cholesterol (mg/dl)	144.05 ± 19.34	140.02 ± 20.28	−4.05	132.21 ± 17.28	123.72 ± 14.78	−8.50	.3885
LDL‐C/HDL‐C	2.52 ± 0.89	2.45 ± 0.91	−0.07	2.36 ± 0.92	2.19 ± 0.99	−0.16	.3462
Renal and hepatic markers							
AST (IU/L)	21.25 ± 15.11	20.36 ± 3.93	−1.02	26.26 ± 7.77	25.28 ± 9.77	−1.06	.4658
ALT (IU/L)	27.72 ± 11.61	26.63 ± 13.4	−2.42	26.94 ± 17.44	26.51 ± 12.75	0.45	.4987
BUN (mg/dl)	15.03 ± 4.45	14.32 ± 3.48	−0.17	12.25 ± 2.13	12.99 ± 3.02	0.79	.1827
Cre (mg/dl)	0.68 ± 0.13	0.67 ± 0.14	−0.01	0.70 ± 0.15	0.71 ± 0.16	0.01	.3879

### 3.3 Effects of sudachi extract on markers of subcutaneous and visceral fat

The baseline parameters for subcutaneous and visceral fat area assessed by CT analysis were comparable between the two study groups. Significant differences were observed in the net changes in ratio of subcutaneous fat area (SFA) to visceral fat area (VFA) (−0.0265%, *p* = .0485), and VFA and waist circumference were also decreased in the sudachi extract group (*p* = .0639 and *p* = .0613, respectively) (Table 3). There was a reduction in BP within the sudachi extract group, but no significant change in the placebo group, suggesting the potential ability of sudachi extract to improve BP.

**TABLE 3:** Change of body composition and blood pressure

	Sudachi			Placebo			*t*‐test
	Baseline	12 weeks	Change	Baseline	12 weeks	Change	
Body fat analysis by CT							
TFA (cm^2^)	243.7 ± 63.9	241.2 ± 60.2	−2.48	281.2 ± 72.8	289.6 ± 71.0	8.27	.176
SFA (cm^2^)	161.6 ± 49.5	161.7 ± 63.6	0.11	186.7 ± 51.3	190.6 ± 61.3	3.87	.534
VFA (cm^2^)	82.1 ± 32.3	79.7 ± 41.0	−2.45	94.6 ± 29.7	99.0 ± 31.4	4.44	.064
VFA/SFA	0.56 ± 0.04	0.54 ± 0.03	−0.027	0.55 ± 0.05	0.56 ± 0.04	0.01	.049
Waist circumference (cm)	89.7 ± 6.2	88.7 ± 6.8	−0.95	91.8 ± 5.1	92.50 ± 5.4	0.67	.061
Blood pressures (BP, mm Hg)							
Systolic BP	134.4 ± 19.4	132.1 ± 18.2	−2.2	134.0 ± 15.8	136.1 ± 16.5	0.110	.372
Diastolic BP	80.2 ± 10.8	77.2 ± 10.9	−3.2	82.4 ± 9.9	83.94 ± 10.18	−0.14	.109
Hypertension (>140/90 mm Hg)	3	3	0	4	4	0	*n*.s
Heart rate (bpm)	71.2 ± 10.3	70.25 ± 10.1	−1.3	71.1 ± 11.2	69.16 ± 11.3	−1.5	.983

### 3.4 Subgroup analysis

Since no significant interaction was observed between sex and age, combined results were reported for men and women. Although there were no significant differences between the two groups when Stratified analysis was performed in subjects with triglycerides (TG) >140 mg/dl, HbA1c >6.0%, or waist circumference >100 cm. On contrast, when Stratified analysis was performed in subjects with visceral fat area (VFA) >100 cm^2^, total fat area (TFA), VFA, subcutaneous fat area (SFA), and VFA/SFA are reduced in sudachi extract group (Table 4). In addition, LDL cholesterol and ratio of LDL cholesterol to HDL cholesterol (*p* < .001).

**TABLE 4:** Subgroup analysis in subject with large VFA

	Sudachi			Placebo		
	Baseline	12 weeks	*p* Wilcoxon	Baseline	12 weeks	*p* Wilcoxon
Markers on glycemic control						
HbA1c (%)	5.86 ± 0.70	5.84 ± 0.62	.699	5.70 ± 0.36	5.67 ± 0.37	.187
Fasting glucose (mg/dl)	90.9 ± 11.5	94.1 ± 8.4	.384	97.5 ± 13.1	97.5 ± 10.9	.709
Fasting insulin (uU/ml)	6.61 ± 1.9	7.08 ± 2.0	.265	6.21 ± 3.1	7.85 ± 4.3	.324
HOMA IR	1.48 ± 0.44	1.67 ± 0.51	.438	1.54 ± 0.86	1.87 ± 0.97	.349
HOMA β	101.5 ± 54.4	84.9 ± 27.6	.453	66.3 ± 34.1	88.6 ± 56.8	.785
Lipid profile						
Triglyceride (mg/dl)	118.0 ± 34.1	133.4 ± 56.7	.185	126.5 ± 39.4	134.7 ± 72.4	.181
Total cholesterol (mg/dl)	214.3 ± 35.9	210.3 ± 36.0	<−0.05	242.5 ± 20.5	237.04 ± 18.1	.079
HDL cholesterol (mg/dl)	52.9 ± 14.5	52.1 ± 15.8	.923	55.3 ± 10.5	54.8 ± 7.0	.925
LDL cholesterol (mg/dl)	132.8 ± 31.0	120.9 ± 30.3	<.001	171.0 ± 8.3	166.3 ± 21.5	.282
LDL‐C/HDL‐C	2.67 ± 0.86	2.45 ± 0.75	<.001	3.17 ± 0.51	3.10 ± 0.73	.096
Body fat analysis						
Body fat (%)	30.6 ± 6.2	29.6 ± 6.4	.089	28.9 ± 4.6	28.6 ± 4.6	.903
TFA (cm^2^)	318.8 ± 60.3	309.8 ± 63.1	<.001	313.4 ± 41.3	316.3 ± 41.5	.908
SFA (cm^2^)	194.7 ± 60.0	188.9 ± 57.8	<.001	179.3 ± 41.0	182.6 ± 36.6	.964
VFA (cm^2^)	124.1 ± 14.5	121.0 ± 23.6	<.001	134.1 ± 11.7	133.7 ± 24.1	.884
VFA/SFA	0.68 ± 0.20	0.58 ± 0.21	<.001	0.79 ± 0.27	0.76 ± 0.26	.853
Waist circumference (cm)	95.3 ± 4.7	94.5 ± 4.8	.058	98.9 ± 7.1	98.1 ± 5.4	.855
Blood pressures (BP, mm Hg)						
Systolic BP	135.1 ± 15.2	140.8 ± 8.3	.373	145.8 ± 25.8	142.7 ± 19.9	.817
Diastolic BP	82.8 ± 10.8	85.8 ± 10.7	.583	91.0 ± 13.9	88.0 ± 10.1	.678
Hypertension (>140/90 mm Hg)	2	2	‐	3	3	‐
Heart rate (bpm)	71.4 ± 8.7	70.9 ± 8.9	.913	70.8 ± 11.2	69.8 ± 11.5	.918

### 3.5 Adverse events

We documented no adverse events, with no dark stool, diarrhea, abnormal pain, or dizziness reported by any of the participants. No participants withdrew from the trial due to adverse events.

### 3.6 Compliance

By counting the remaining capsules at every visit, the compliance of participants was assessed in both intervention groups. The actual capsule consumption rates (accounting for total provided capsules) were 95.5% in the placebo group and 92.2% in the sudachi group.

## 4 DISCUSSION

To our knowledge, this is the first RCT specifically conducted among Japanese middle‐aged men and women with individuals at risk for developing diabetes to investigate the effects of sudachi extract powder on glucose homeostasis, insulin sensitivity, and lipid profile. The results indicated that a 12‐week course of sudachi extract produced favorable reductions in visceral fat. Subgroup analysis indicated that patients with elevated metabolic markers may obtain the additional benefit of improvements in lipid profiles, however, there were no significant differences in any glycemic parameters at any observation point, suggesting that the number of enrolled subjects in this study was too small to confirm this.

The validity of this trial is strengthened by aspects of its design. The trial was randomized and placebo‐controlled and was able to provide the first level of scientific evidence. We strictly followed the principle of random allocation and allocation concealment. The well‐balanced baseline characteristics demonstrated successful randomization. The study was conducted among participants with individuals at risk for developing diabetes, which eliminated the possible masking effect or interactions of sudachi and medications. We assessed the effect of sudachi peel extract powder on whole‐body glucose and lipid homeostasis. Participants maintained their usual dietary habits and physical activity patterns throughout the follow‐up, which minimized possible bias from lifestyle modifications, and this allowed us to infer an independent effect of sudachi peel extract powder on metabolic profiles.

Sudachi peel includes several functional components, including hesperidin and sudachitin, and there are several studies that have reported on the benefits of components in citrus peel for metabolic syndrome. Hesperidin is a flavone glycoside found in abundance in the peel of citrus fruits. In rats, hesperidin improved hypercholesterolemia, hypertriglyceridemia, and fatty liver. (Akiyama et al., 2009; Wang et al., 2011) Morand et al. showed that intake of orange juice including hesperidin decreased diastolic BP and, postprandially, endothelium‐dependent microvascular reactivity. (Morand et al., 2011) The dose of active ingredient in their RCT included 292 mg/day of hesperidin whereas our study provided only 12.5 mg/day (File S1); therefore, our results cannot be supported by the effect of hesperidin. Sudachi peel also includes narirutin and naringin, although their concentrations in our extract were 0.7% and 0.5%, respectively. Naringenin has been shown to improve obesity‐related diseases in mice. (Assini et al., 2013; Mulvihill et al., 2010) The capsules in this study contained 1.4% sudachitin, making the daily dose greater than that in the study by Morand et al. Although its concentration is similar to that of hesperidin, it could be that there is an interaction between hesperidin and sudachitin or that sudachitin is effective at lower concentrations. Sudachitin is composed of 4′,5,7‐trihydroxy‐3′,6,8‐trimethoxyflavone and 3′‐demethoxysudachitin. These compounds were identified in sudachi peel as having the greatest antimicrobial activity against methicillin‐resistant *Staphylococcus* *aureus* and *Helicobacter* *pylori*. (Nakagawa et al., 2006) In addition, Yuasa et al. showed that sudachitin inhibited nitric oxide production by suppressing the expression of inducible nitric oxide synthase, indicating that sudachitin had an anti‐inflammatory effect. (Yuasa et al., 2012) Furthermore, we previously clarified that sudachitin increased mitochondrial function in skeletal muscle resulting in enhancement of energy expenditure in mice. (Tsutsumi et al., 2014) Administration of 2 mg kg day^‐1^ sudachitin for 12 weeks decreased TG levels, had an antiobesity effect, and improved glucose tolerance in high‐fat diet‐induced obesity in mice. Several studies have been published on nobiletin, a methoxyflavone whose structure is similar to that of sudachitin. Mulvihill et al. showed that nobiletin (20 mg kg^‐1^ day^‐1^ over 12 weeks) attenuated dyslipidemia and atherosclerosis in obese mice. (Mulvihill et al., 2011) The concentration of sudachitin in our study was only 1.4%, and this might explain why the only statistically significant effect of sudachi peel seen was a reduction in the ratio of visceral fat to subcutaneous fat; it could be that the concentration was too low to make other effects significant. In this study, we set the amount of sudachitin provided to the subjects as 4.9 mg, which was equivalent to that of 1/3 of a whole Citrus sudachi which is a very small amount but confirmed the safety in previous study (Akaike et al., 2014). On the contrary, in mice, the daily sudachitin intake is 20 mg/kg bodyweight, which is a very high dose. This was set to an amount that was effective when many flavonoids were administered to mice, considering that mice have different metabolic rates from humans.

In the present trial, we observed a modest reduction in visceral fat with the sudachi extract treatment, but null effects on glucose and insulin. Visceral fat reflects the average lipid profile level during the past 2–3 months, and has the advantage of much lower variability compared to glucose and insulin, allowing small treatment differences to be detected. (Barua et al., 2014) Evidence has shown that visceral fat measurements alone are sufficient to provide an accurate estimate of metabolic syndrome, FG as early as within 4 weeks of starting antidiabetic therapy. (Barua et al., 2014) This could explain the significant effect of sudachi extract on lipid profile in diabetes models but the nonsignificant effect in our participants.

### 4.1 Strengths and limitations of this study

For the first time, we found that sudachi peel extract powder improves lipid profile and glucose metabolism. A randomized, double‐blind clinical trial was conducted to investigate the benefit of sudachi peel extract powder. Intake of sudachi peel extract powder improved the ratio of visceral fat to subcutaneous fat.

On the other hand, this study has several limitations. First, we only found favorable changes in the ratio of visceral fat to subcutaneous fat, but not in other markers. This could be explained by the small number of participants. Although 88 healthy people enrolled, only 41 met our inclusion condition of BMI 23–30 kg/m^2^. Since we did not see significant interaction between sex and age, the results were reported combined for men and women. However, the participants with high VFA were only men; therefore, it could be that sudachi peel extract is more effective at reducing visceral fat in men than in women.

Second, like most other RCTs using flavonoids as a treatment, we would not have been able to detect sudachitin or the metabolites of its constituents in serum samples as a measure of compliance, mainly due to the short half‐life of sudachitin (2 hr); the 8‐ to 10‐hr fasting period before blood collection resulted in complete clearance and excretion of sudachitin.

Another limitation of this work was possible incomplete blinding in the placebo group, which may have resulted in two participants dropping out. Since this would produce conservative results for the active treatment we conclude that the early dropouts from the placebo group would have been unlikely to affect the findings.

Finally, the dose–response relationship was not tested in our study. In addition, the relatively short duration of our trial may account for the nonsignificant findings in insulin resistance. Future studies of longer duration are warranted since an increased length of treatment may achieve statistical and clinical relevance, especially for the modest efficacy of treatment typically achieved with phytochemicals.

## 5 CONCLUSIONS

In conclusion, the 12‐week RCT in Japanese adults with individuals at risk for developing diabetes indicated that purified sudachi has beneficial effects on accumulation of visceral fat. Future studies of longer duration that explore the dose–response relationship among patients with overweight are warranted to confirm our findings.

## CONFLICT OF INTEREST

All supplements used in this study were provided by Ikeda Yakusou.

## ETHICAL APPROVAL

This study was approved by the Institutional Review Board of Linking Setouchi Innate immune network.

## INFORMED CONSENT

Written informed consent was obtained from all study participants.

## Supporting information

File S1
