# Supplementary material for: Sudachi peel extract powder including the polymethoxylated flavone sudachitin improves visceral fat content in individuals at risk for developing diabetes
Source: Food Sci Nutr. 2021 Jun 15;9(8):4076–84. doi: 10.1002/fsn3.2339 (PMC8358332; doi:10.1002/fsn3.2339)
Supplement: Supplementary file 1 — File S1 [file FSN3-9-4076-s001.docx]

Supplementary file

Composition of sudachi extract powder

|  | dairy intake (mg) |
| --- | --- |
| 1.4% Sudachitin | 4.9 |
| 3.7% Hesperidin | 12.95 |
| 0.7% Narirutin | 2.45 |
| 0.5% Naringenin | 1.75 |

Other (including fiber) 327.95

350mg/day
